# Bacterial Genomics Reveal the Complex Epidemiology of an Emerging Pathogen in Arctic and Boreal Ungulates

**DOI:** 10.3389/fmicb.2016.01759

**Published:** 2016-11-07

**Authors:** Taya L. Forde, Karin Orsel, Ruth N. Zadoks, Roman Biek, Layne G. Adams, Sylvia L. Checkley, Tracy Davison, Jeroen De Buck, Mathieu Dumond, Brett T. Elkin, Laura Finnegan, Bryan J. Macbeth, Cait Nelson, Amanda Niptanatiak, Shane Sather, Helen M. Schwantje, Frank van der Meer, Susan J. Kutz

**Affiliations:** ^1^Faculty of Veterinary Medicine, University of CalgaryCalgary, AB, Canada; ^2^Institute of Biodiversity, Animal Health and Comparative Medicine, University of GlasgowGlasgow, UK; ^3^Alaska Science Center, U.S. Geological SurveyAnchorage, AK, USA; ^4^Environment and Natural Resources, Government of Northwest TerritoriesInuvik, NT, Canada; ^5^Department of Environment, Government of NunavutKugluktuk, NU, Canada; ^6^Environment and Natural Resources, Government of Northwest TerritoriesYellowknife, NT, Canada; ^7^fRI ResearchHinton, AB, Canada; ^8^Ministry of Forests, Lands and Natural Resource Operations, Government of British ColumbiaNanaimo, BC, Canada; ^9^Department of Environment, Government of NunavutCambridge Bay, Nunavut, Canada; ^10^Canadian Wildlife Health CooperativeCalgary, AB, Canada

**Keywords:** bacteria, emerging disease, *Erysipelothrix*, genomics, molecular epidemiology, ungulate, wildlife

## Abstract

Northern ecosystems are currently experiencing unprecedented ecological change, largely driven by a rapidly changing climate. Pathogen range expansion, and emergence and altered patterns of infectious disease, are increasingly reported in wildlife at high latitudes. Understanding the causes and consequences of shifting pathogen diversity and host-pathogen interactions in these ecosystems is important for wildlife conservation, and for indigenous populations that depend on wildlife. Among the key questions are whether disease events are associated with endemic or recently introduced pathogens, and whether emerging strains are spreading throughout the region. In this study, we used a phylogenomic approach to address these questions of pathogen endemicity and spread for *Erysipelothrix rhusiopathiae*, an opportunistic multi-host bacterial pathogen associated with recent mortalities in arctic and boreal ungulate populations in North America. We isolated *E. rhusiopathiae* from carcasses associated with large-scale die-offs of muskoxen in the Canadian Arctic Archipelago, and from contemporaneous mortality events and/or population declines among muskoxen in northwestern Alaska and caribou and moose in western Canada. Bacterial genomic diversity differed markedly among these locations; minimal divergence was present among isolates from muskoxen in the Canadian Arctic, while in caribou and moose populations, strains from highly divergent clades were isolated from the same location, or even from within a single carcass. These results indicate that mortalities among northern ungulates are not associated with a single emerging strain of *E. rhusiopathiae*, and that alternate hypotheses need to be explored. Our study illustrates the value and limitations of bacterial genomic data for discriminating between ecological hypotheses of disease emergence, and highlights the importance of studying emerging pathogens within the broader context of environmental and host factors.

## Introduction

Emerging infectious diseases are increasingly reported in wildlife, often in association with changing environmental conditions (Daszak et al., 2000). Environmental change may alter the community structure of hosts or pathogens, including species diversity, composition and contact patterns, and may also influence host-parasite interactions and disease dynamics (Gottdenker et al., 2014; Van Hemert et al., 2015). Northern ecosystems are changing especially rapidly, where unprecedented rates of climate change are influencing a wide range of ecological processes (Post et al., 2013; IPCC, 2014). Several recent emergence events of macroparasites in northern wildlife have been linked to climate warming (Kashivakura, 2013; Kutz et al., 2013; Laaksonen et al., 2015), whereas the role of climate change in emerging diseases caused by microparasites, including viruses, bacteria and protozoa, is not yet as clearly established (e.g., Goldstein et al., 2009; Kutz et al., 2009; Harms, 2012; Loiseau et al., 2012; Ducrocq et al., 2013; Hansen et al., 2015; but see Ytrehus et al., 2015). Pathogen emergence poses a risk to the health and sustainability of wildlife populations which, in addition to their intrinsic value and ecosystem services, continue to be an important component of community health and food security for many indigenous communities in temperate and arctic regions of North America (Inuit Circumpolar Council- Canada, 2012; Council of Canadian Academies, 2014).

The mechanisms behind the emergence of pathogens or novel disease presentations in previously unaffected ecosystems are often difficult to establish. One of the key questions associated with emergence events is whether they are caused by new introductions, or whether they are outbreaks of endemic pathogens. The ability to make such distinctions is often hampered by a paucity of baseline data on pathogen biodiversity, especially in ecosystems poorly characterized in this respect, such as those at northern latitudes (Hoberg et al., 2008; Kutz et al., 2009, 2014; Van Hemert et al., 2014).

Recently, widespread mortality events of muskoxen (*Ovibos moschatus wardi*) in the Canadian Arctic Archipelago associated with *Erysipelothrix rhusiopathiae* were reported (Kutz et al., 2015). This was the first report of *E. rhusiopathiae* in the Arctic, and the first time *E. rhusiopathiae* was associated with large-scale ungulate mortalities. *Erysipelothrix rhusiopathiae* is a Gram positive, facultative intracellular bacterium, best known as an opportunistic pathogen of swine (Opriessnig and Wood, 2012), poultry (Bricker and Saif, 2013), and humans (Veraldi et al., 2009). It is a ubiquitous, globally-distributed multi-host opportunistic pathogen and commensal which has been found in terrestrial and aquatic mammals, birds, fishes and arthropods (Brooke and Riley, 1999). Reports of *E. rhusiopathiae* causing disease in wildlife have been sporadic (Leighton, 2008). Rare outbreaks with a large number of mortalities have involved avian hosts (Wolcott, 2007), or mice (Wayson, 1927), whereas mortalities in wild ungulates were previously limited to small numbers (Bruner et al., 1984; Campbell et al., 1994). Both cattle (Hassanein et al., 2003; Zambrano-Nava et al., 2011) and swine (Stephenson and Berman, 1978) can act as asymptomatic carriers, with *E. rhusiopathiae* isolated from tonsils, bone marrow (Spears, 1955), and other tissues at slaughter of apparently healthy animals, and it is also part of the normal vaginal flora of cattle (Zambrano-Nava et al., 2011). *Erysipelothrix rhusiopathiae* appears to have a particular tropism for tonsils; tonsillar crypts are a likely port of entry and site for persistent infection in swine (Harada et al., 2013). It is probable that this bacterium is carried by various additional host species, including wildlife, with no clinical signs (Wang et al., 2010), although there is little empirical evidence available to support this hypothesis. Transmission of *E. rhusiopathiae* is thought to occur primarily through indirect contact with bacteria shed in feces, urine, saliva and nasal secretions; it is also hypothesized that arthropods may occasionally act as vectors (Brooke and Riley, 1999). Although there is currently no evidence that *E. rhusiopathiae* can replicate in the environment (Wood, 1973; Chandler and Craven, 1980), the bacterium can survive for up to several months outside of a host depending on the ambient conditions (Mitscherlich and Marth, 1984). It has been hypothesized that this ability to persist in the environment for extended periods, in addition to *E. rhusiopathiae*'s wide host range, are the most important factors facilitating its widespread distribution (Brooke and Riley, 1999). Several virulence factors have been demonstrated or suggested for this bacterium, including neuraminidase, surface protective antigen A and the capsular polysaccharide (Ogawa et al., 2011; Shi et al., 2012), the majority of which appear to be commonly present (Janßen et al., 2015). Isolates of *E. rhusiopathiae* sequenced to date fall into three distinct clades, all of which appear to have retained some degree of host generalism (Forde et al., 2016), suggesting that most strains can likely be transmitted between species.

*Erysipelothrix rhusiopathiae* septicemia was determined to be the proximate cause of acute deaths among muskoxen of all ages and both sexes on Banks and Victoria Islands, Northwest Territories and Nunavut, Canada, first noted in 2010 (Kutz et al., 2015). These two islands, which encompass an area of over 300,000 km^2^, were previously home to over 70% of muskoxen in Canada (Ferguson and Gauthier, 1991). Their combined population increased from around 75,000 in the late 1980's to approximately 140,000 animals in 2001 (Dumond, 2006). Recent surveys on both islands suggest that these populations have since declined by more than 50% (Davison et al., 2014; Dumond and Leclerc, Government of Nunavut, unpublished data 2014); only on the northwest corner of Victoria Island has the muskox population remained relatively stable since 2005 (Davison and Williams, 2015). The extent to which the *E. rhusiopathiae*-associated mortalities have contributed to these population declines is unknown, however, high mortality rates of adult animals are likely to have had an impact at the population scale.

Over a similar timeframe to the muskox population declines in Nunavut and Northwest Territories, higher than expected mortality rates or unexplained mortalities were observed in other northern ungulate populations. On the Northern Seward Peninsula of Alaska, US, mortality rates of 14–33% were recorded among adult radio-collared muskoxen June through August 2009–12 (L. G. Adams, U.S. Geological Survey, unpublished data). In northeastern British Columbia (BC), Canada, during a broad study on the health of boreal caribou populations (*Rangifer tarandus caribou*) the annual survival rate among radio-collared adult females between May 2013 and April 2014 was lower than expected (Culling and Culling, 2014). Multiple deaths among caribou were observed in this study area between December 2012 and July 2013 that could not be attributed to predation (B. Macbeth, University of Calgary, unpublished data). Meanwhile, in central BC, population declines in moose (*Alces alces*) of 50–70% were noted over a 7–15 year period (BC FLNRO, 2012, 2015; Kuzyk and Heard, 2014). These observations of higher than expected mortality of unknown etiology in muskoxen in Alaska and boreal caribou in BC similar to that observed among muskoxen on Banks and Victoria Islands, along with declining moose populations in parts of BC—all across a similar timeframe—led us to hypothesize that these mortalities might be part of a common emerging disease event associated with *E. rhusiopathiae*. Given its previously documented effects on muskoxen (Kutz et al., 2015), the potential role of *E. rhusiopathiae* in these other wild ungulate mortalities warrants investigation.

The aims of this study were to (1) test for the presence of *E. rhusiopathiae* in geographically disparate ungulate populations across northern latitudes of North America, and (2) quantify molecular diversity and evolutionary relationships among isolates to assess if recent disease events and population declines are associated with endemic or recently introduced pathogens, and whether a single strain was involved in the disparate mortalities. We took a bacterial genomic approach to achieve the maximum level of resolution for investigating genetic diversity and relatedness among strains (Robinson et al., 2013; Kao et al., 2014) in order to gain insights into the processes underlying the possible emergence of *E. rhusiopathiae* in northern wild ungulates.

## Materials and methods

### Samples

Samples were obtained for *E. rhusiopathiae* testing from a variety of sources as part of ongoing research and monitoring activities across northern North America. Unless otherwise stated, samples were frozen at −20°C until DNA extraction and bacterial culture in 2013–2014.

#### Sample group 1: muskoxen in the canadian arctic archipelago

**Figure 1:**
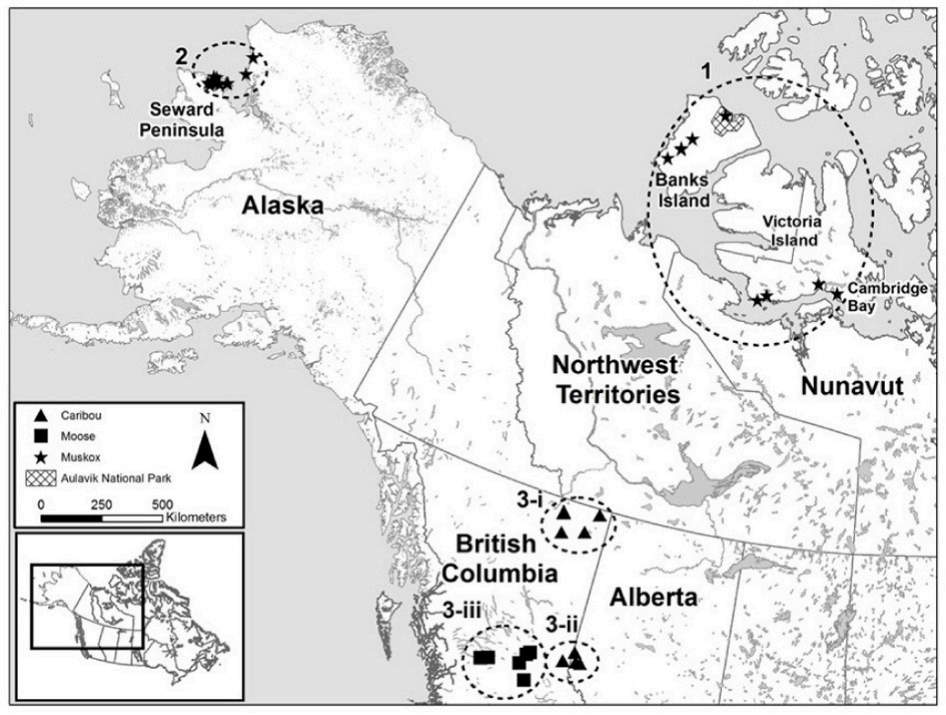
**Map of sampling sites of ungulate carcasses from across northern North America tested for the presence and diversity of *Erysipelothrix rhusiopathiae***. Samples were collected from muskoxen (*Ovibos moschatus wardi*) on Banks and Victoria Islands (Sample Group 1) and in northwestern Alaska (Sample Group 2), from boreal caribou (*Rangifer tarandus caribou*, Designatable Unit 6) in northeastern British Columbia (Sample Group 3-i), central mountain caribou (*R. t. caribou*, Designatable Unit 8) in western Alberta (Sample Group 3-ii), and from moose (*Alces alces*) in central British Columbia (Sample Group 3-iii).

**Figure 2:**
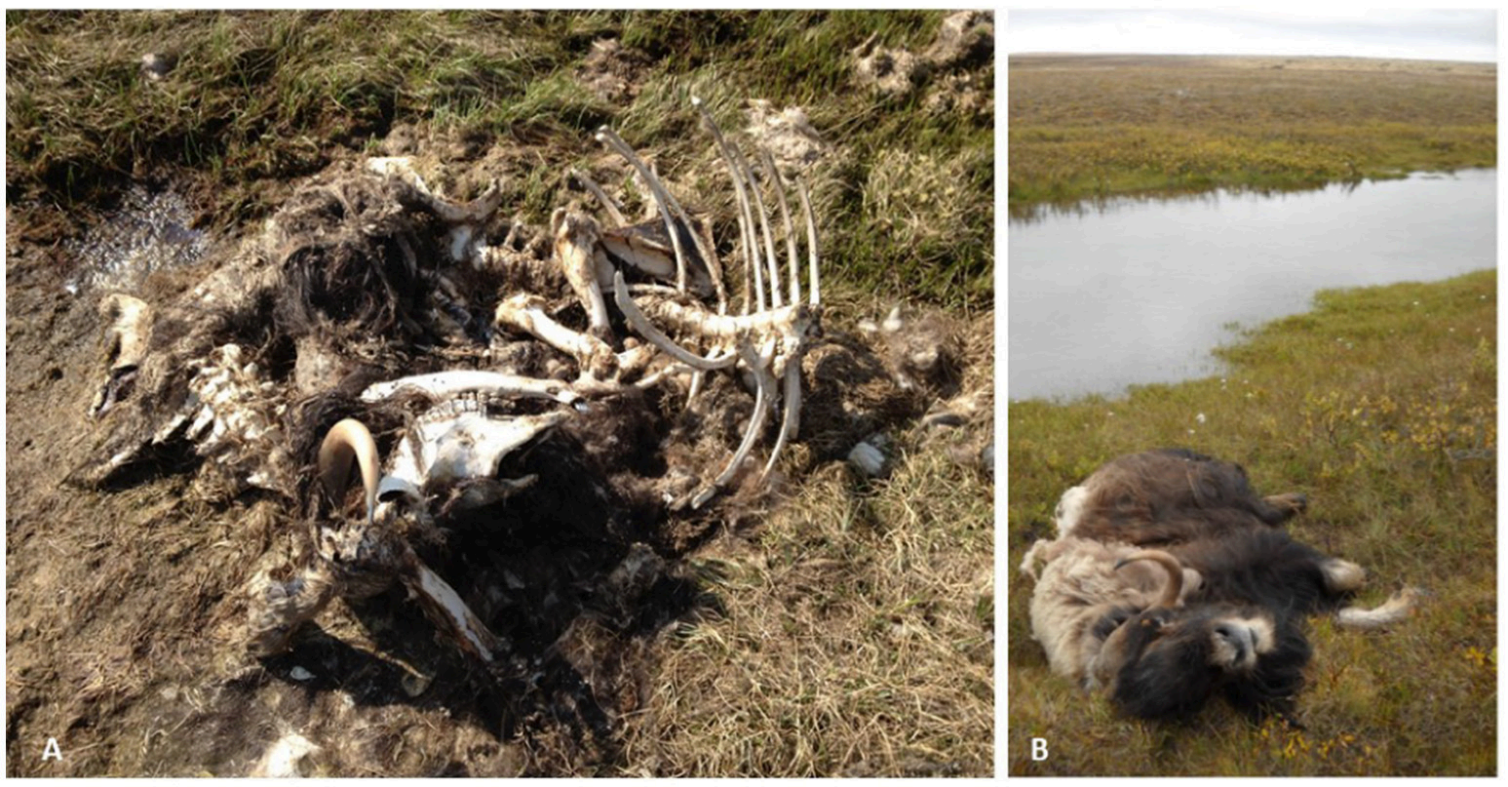
**Carcass sites of AMX7 (A) from Aulavik National Park on northern Banks Island, and MX10-3 (B) near Wellington Bay on Victoria Island**. The muskox carcasses found on Victoria Islands in 2010 and 2011 and on Banks Island in 2012 were animals in good body condition that appeared to have suffered an acute death (Kutz et al., 2015). *Erysipelothrix rhusiopathiae* was isolated from the bone marrow of AMX7 despite the advanced state of decomposition.

Tissue and/or fecal samples were collected from 19 muskox carcasses during the widespread mortality events on Banks and Victoria Islands (Table 1): six on southwestern Banks Island between July and August 2012 (one of which was found moribund and was euthanized), four near Wellington Bay on Victoria Island in August 2010, and three east of Lady Franklin Point on Victoria Island in August 2011 (Figure 1). Long bones were collected from six carcasses in advanced states of decomposition found in Aulavik National Park on northern Banks Island in June 2013 (Figures 1, 2A). Pathological findings of these carcasses are detailed in Kutz et al. (2015). To determine if muskoxen could be asymptomatic carriers of *E. rhusiopathiae*, additional samples were obtained opportunistically from commercial harvests and sport hunts. Tonsils from 60 muskoxen were collected during the commercial harvest near Sachs Harbor on Banks Island in November, 2012. Additionally, tonsils (*n* = 179) and lymph nodes (*n* = 66) from 182 individual muskoxen (Table 1) were collected during commercial harvests near Cambridge Bay on Victoria Island over three consecutive years (2010–2012). Metatarsal bones (*n* = 19) were collected from sport hunted muskoxen on Victoria Island in August 2014.

**Table 1:** **Type and number of samples tested for *Erysipelothrix rhusiopathiae* from various wild ungulate populations sampled across northern North America between 2010 and 2014**.

**Sample group**	**Species**	**Location**	**Sample type**	**Collection date**	**# Samples (# Animals) tested**	**# qPCR positive/tested**	**# Culture positive/tested**	**# Isolates sequenced (# Animals)^a^**
1	Muskox	Banks Island	Various tissues; carcasses	Jul–Aug 2012	17 (6)	13/16	17/17	23 (6)
1	Muskox	Banks Island (Aulavik)	Long bones (marrow); carcasses	Jul 2013	10 (6)	4/10	1/10	1 (1)
1	Muskox	Victoria Island	Tissues and/or feces; carcasses	Aug 2010	12 (4)	10/12	8/12	9 (3)
				Aug 2011	12 (3)	12/12	10/12	10 (3)
1	Muskox	Banks Island	Tonsils; commercial harvest	Nov 2012	60	0/60	–	–
1	Muskox	Victoria Island	Tonsils (T) ± lymph nodes (LN); commercial harvest	Feb–Mar 2010	66T	3/66	1/3	2 (1)
				Feb–Mar 2011	72T; 66LN (75)	1/138	0/21	–
				Feb–Mar 2012	41T	0/41	0/36	–
1	Muskox	Victoria Island	Metatarsal marrow; sport hunt	Aug 2014	19	0/19	0/19	–
2	Muskox	Alaska	Bone marrow; carcasses	Nov 2010–Sep 2012	26 (23)	12/26	6/26	11 (6)
3 (i)	Caribou (boreal DU 6)	British Columbia	Various tissues, bone marrow and blood; carcasses	2013–2014	40 (16)	3/32	10/40	13 (7)
3 (ii)	Caribou (central mountain DU 8)	Alberta	Various tissues, bone marrow and blood; carcasses	2013–2014	23 (9)	2/20	7/23	9 (6)
3 (iii)	Moose	British Columbia	Bone marrow; carcasses	2014	22	3/22	7/22	9 (7)
Total					486 (350)	63/474	67/241	87 (40)

Samples are grouped broadly by species and geographic location, and correspond to those in Figure 1.

a In some cases, multiple isolates were sequenced from a single tissue. DU, Designatable Unit; PCR, polymerase chain reaction.

#### Sample group 2: muskoxen in Alaska

Intact long bones or jaw bones were collected from carcasses of radio-collared muskoxen that died between November 2010 and September 2012 in the Northern Seward Peninsula (*n* = 16) and Cape Thompson (*n* = 7) populations of northwestern Alaska. These populations cover areas of 12,200 and 10,800 km^2^, respectively. Bones were retrieved several months to more than a year after the time of death.

#### Sample group 3: caribou and moose on the Northern Canadian Mainland

(i) Various tissue and bone marrow samples (Table 1) were collected from 16 boreal caribou from northeastern BC (Designatable Unit (DU) 6, representing a distinct management unit for population assessments; (COSEWIC, 2011)). Most samples were collected after an estimated delay of several days to weeks based on collar data or visual appraisal; however, one animal seen moribund was sampled the next day (SKSLP2). (ii) Various tissue and bone marrow samples were collected from central mountain caribou (DU 8) from western Alberta (*n* = 9). These samples were collected across an area encompassing approximately 7400 km^2^, between 1 and 4 weeks after death. (iii) Bone marrow samples from moose carcasses (*n* = 22) from eastern BC were collected in 2014 across an area of approximately 30,000 km^2^. Samples from radio-collared moose (*n* = 6) were collected within 1 week of death; those from un-collared moose (*n* = 3) were estimated to be collected within 1–3 weeks from the time of death. As a preliminary investigation into the host range of *E. rhusiopathiae* on Banks and Victoria Islands, samples were opportunistically collected from species sympatric to muskoxen on these islands and on the nearby mainland (Supplementary File 1, Supplementary Table 1). All sample collections were approved by the University of Calgary Animal Care Committee (AC13-0072), as well as by federal, provincial, territorial and state government agencies where applicable.

### Laboratory methods

The laboratory methods used in this study were previously described in detail (Forde et al., 2016). DNA template was prepared using 2 g of tissue or bone marrow mechanically homogenized in 5 ml of phosphate buffered saline (PBS) using a Stomacher 80 Biomaster (Seward, Port Saint Lucie, FL, USA) on high speed for 3 min. Contents were transferred to a 15 ml Falcon tube and centrifuged at 2500 × g for 30 min; 200 μl of the supernatant was transferred to a 1.5 ml Eppendorf tube, after which the DNeasy Blood and Tissue Kit (Qiagen, Mississauga, ON, Canada) was used starting at step 2. To yield a higher DNA concentration than normally specified, 50 μl of the elution buffer provided was used for the final step. For DNA extraction from feces (200 mg), the QIAamp DNA Stool Mini Kit was used following manufacturers' instructions (Qiagen). Quantitative polymerase chain reaction (qPCR) using *E. rhusiopathiae*-specific primers and probe was performed on DNA extracts (Pal et al., 2010).

For the culture of *E. rhusiopathiae* from carcasses, 2 g of tissue or feces were mechanically homogenized in 20 ml of brain heart infusion (BHI) broth with 5% fetal bovine or horse serum, incubated overnight at 37°C with 5% CO_2_, followed by 48 h incubation in selective medium containing kanamycin, neomycin and vancomycin, based on BHI broth with 5% serum (Wood, 1965; Bender et al., 2009); all sample homogenates were also diluted 1:10. Both dilutions were sub-cultured to agar plates of the same selective medium and incubated for 48–72 h at 37°C with 5% CO_2_. Colonies were further sub-cultured to Columbia Agar (CA) with 5% sheep blood (BD-Canada, Mississauga, ON, Canada) for morphological characterization; from these plates, a single colony was sub-streaked to a new CA plate for DNA extraction using the DNeasy Blood and Tissue Kit as previously described. DNA extracts from clonal bacterial populations were confirmed to be *E. rhusiopathiae* using qPCR. Any samples with colony morphology consistent with *Erysipelothrix* spp. (*E. rhusiopathiae* and *E. tonsillarum* are indistinguishable) but which tested negative using *E. rhusiopathiae*-specific qPCR were subsequently tested using the same qPCR reaction but with a probe specific for *E. tonsillarum* (Pal et al., 2010; Supplementary File 2). At least one isolate from each *E. rhusiopathiae* culture-positive sample was sequenced, and in a subset of samples where positive results were obtained for both dilutions, multiple isolates were sequenced from a single tissue (Table 2). DNA was prepared for sequencing using the Nextera XT v2 kit (Illumina, San Diego, CA) and sequenced at the University of Calgary using the Illumina MiSeq platform, generating 250 base pair, paired-end reads.

**Table 2:** ***Erysipelothrix rhusiopathiae* isolates identified within wild ungulate populations sampled across northern North America between 2010 and 2014**.

**Animal ID**	**Species**	**Date sampled (month-year)**	**Location**	**Feces**	**Heart**	**Ileum**	**Kidney**	**Liver**	**Lymph node**	**Lung**	**Marrow**	**Muscle**	**Spleen**	**Tonsil**	**Total**
**SAMPLE GROUP 1**															
172	Muskox	July-12	Banks								1		2		3
173	Muskox	July-12	Banks								1		1		3^#^
174	Muskox	July-12	Banks				1	1			1		1		4
969	Muskox	Aug-12	Banks				1	1					2		4
971	Muskox	Aug-12	Banks				2	1					2		5
972	Muskox	Aug-12	Banks				1	1					2		4
AMX7^*^	Muskox	July-13	Banks								1				1
MX10-1	Muskox	Aug-10	Victoria	1		1			PLN	1					4
MX10-2	Muskox	Aug-10	Victoria							1					1
MX10-3^**^	Muskox	Aug-10	Victoria						MLN, PSLN	2					4
MX11-1	Muskox	Aug-11	Victoria	1	1	1				1					4
MX11-2	Muskox	Aug-11	Victoria	1		1		1	MLN	1					5
MX11-3	Muskox	Aug-11	Victoria	1											1
VI10-23	Muskox	Mar-10	Victoria											2	2
**SAMPLE GROUP 2**															
AKM1	Muskox	Aug-12	AK-NSP								1				1
AKM3	Muskox	Aug-12	AK-NSP								6				6
AKM7	Muskox	Aug-12	AK-CT								1				1
AKM10	Muskox	May-11	AK-NSP								1				1
AKM14	Muskox	July-11	AK-NSP								1				1
AKM19	Muskox	May-11	AK-NSP								1				1
**SAMPLE GROUP 3 (I)**															
SK69	Caribou	May-13	BC								1				1
SKSLP2^*^	Caribou	April-13	BC					1		2	1	1			5
SK11	Caribou	Sept-13	BC								1				1
SK18	Caribou	April-14	BC								2				2
SK106	Caribou	July-13	BC									1			1
SK133	Caribou	July-13	BC									1			1
BC1015	Caribou	Feb-14	BC								2				2
**Sample Group 3 (II)**															
F440^+^	Caribou	May-13	AB								3				3
F446	Caribou	May-13	AB								1				1
F786	Caribou	Oct-13	AB								1				1
F793	Caribou	May-13	AB								1				1
Grizzly	Caribou	Sept-14	AB								1				1
2197	Caribou	May-14	AB								2				2
**SAMPLE GROUP 3 (III)**															
AN540	Moose	Feb-14	BC								1				1
AN541	Moose	Feb-14	BC								2				2
AN551	Moose	Mar-14	BC								1				1
AN557	Moose	Unk-14	BC								1				1
AN562	Moose	April-14	BC								2				2
AN569	Moose	Jun-14	BC								1				1
AN570	Moose	Mar-14	BC								1				1

Sample Groups and locations are shown in Figure 1. The number of isolates sequenced from each individual are shown, including a breakdown by sample type.

AB, Alberta, Canada; AK-CT, Cape Thompson, Alaska, US; AK-NSP, Northern Seward Peninsula, Alaska, US; Banks, Banks Island, Northwest Territories, Canada; BC, British Columbia, Canada; Victoria = Victoria Island, Nunavut, Canada; MLN, mesenteric lymph node; PLN, pharyngeal lymph node; PSLN, pre-scapular lymph node.

* Technical replicate: isolates were re-cultured from frozen glycerol stock, re-extracted, and re-sequenced during a later run.

** Technical replicates performed on two isolates.

# One isolate was provided directly by the Canadian Wildlife Health Cooperative; the tissue of origin is unknown.

+ 2 different marrow samples.

### Bioinformatic analyses

*De novo* assembly and reference-based variant calling were performed as previously described (Forde et al., 2016). To summarize, reads were trimmed and duplicate reads removed using ConDeTri (Smeds and Künstner, 2011) then assembled into contigs with SPAdes (Bankevich et al., 2012), using a *k*-value of 55. Contig improvements were made using the PAGIT suite of programs (Swain et al., 2012). *De novo* assemblies of each isolate were annotated using RAST (Overbeek et al., 2014). To place the newly assembled ungulate isolates within the global phylogeny of *E. rhusiopathiae* (Forde et al., 2016), amino acid fasta files of coding sequences predicted by RAST were used as the input for PhyloPhlAn (Segata et al., 2013). The phylogeny included 150 *Erysipelothrix* spp. isolates, 60 of which were newly described in this publication. To examine the relationship among isolates within the dominant Clade 3 (Forde et al., 2016), a core alignment of the *de novo* assemblies was generated using Parsnp (Treangen et al., 2014). Single nucleotide polymorphisms (SNPs) inferred to be outside of areas of recombination by the program Gubbins (Croucher et al., 2015) were used as the input for PhyML (Guindon and Gascuel, 2003) to estimate a maximum likelihood (ML) tree. We conducted an analysis in PhyML using a generalized time-reversible (GTR) model of nucleotide substitution with a gamma distribution; this was repeated for 1000 bootstrap replicates. Additionally, core genes (those present among all isolates) were visualized using GView (Petkau et al., 2010).

**Figure 3:**
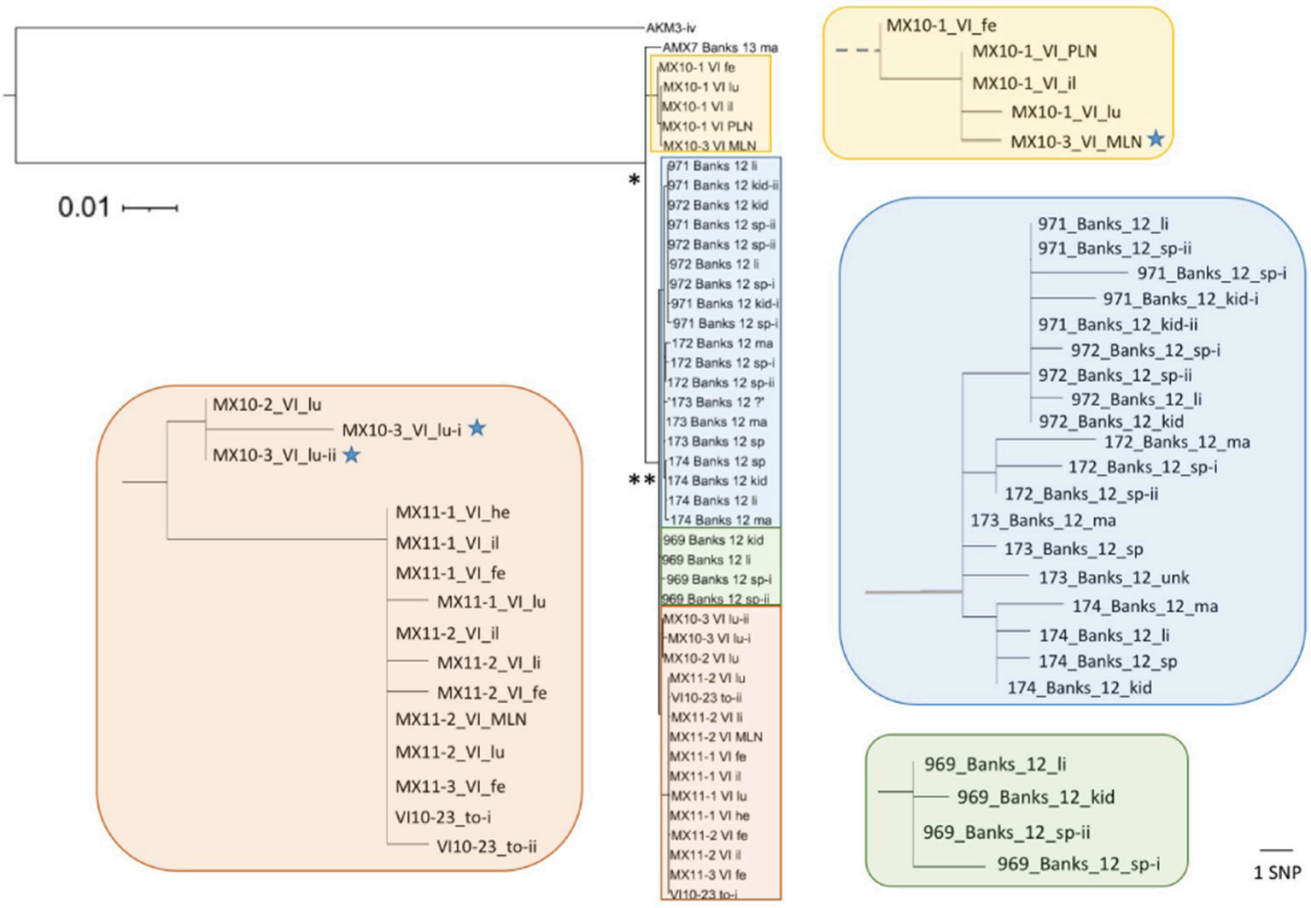
**Diversity of *Erysipelothrix rhusiopathiae* isolates associated with muskox mortalities on Banks and Victoria Islands (VI)**. This maximum likelihood tree (rooted to the most closely related isolate from Alaska muskox AKM3) is based on high quality single nucleotide polymorphisms (SNPs) found taking a reference-based mapping approach. Isolate names include year of isolation, island (Banks or VI) and tissue of origin (fe, feces; il, ileum; kid, kidney; lu, lungs; ma, bone marrow; MLN, mesenteric lymph node; PLN, pharyngeal lymph node; sp, spleen; to, tonsil). Parts of the tree (in colored rectangles) are enlarged to show the distances among closely related isolates. The scale bar to the right shows the branch length equivalent to 1 SNP within these magnified areas of the tree. The inferred common ancestor of all the isolates from the two islands within the clonal lineage (*n* = 44) is shown by (^*^), while the majority of the isolates (*n* = 38) share a more recent common ancestor shown by (^**^). All branches in zoomed boxes originating from this point are shown to scale. Blue stars indicate isolates from MX10-3, from which a more divergent isolate was found in its pre-scapular lymph node (Figure 4B).

For reference-based variant calling, unique, trimmed reads were aligned to the *E. rhusiopathiae* Fujisawa genome [GenBank: NC_015601] (Ogawa et al., 2011) using BWA-MEM with default settings (Li, 2013), and variants called using the mpileup command in SAMtools (Li et al., 2009). To increase the sensitivity for detecting variants between highly similar isolates, slightly lower quality thresholds were implemented than previously described. A list of variant sites was generated across all *E. rhusiopathiae* genomes using custom python scripts using the following parameters: consensus base quality ≥30, mapping quality ≥30, minimum of 2 reads present on each strand, and >95% reads at that site were the majority allele. Alleles at these sites were called for each isolate if at least two high quality reads in any direction were present, and if all reads supported the same allele call. Sites in mobile elements and repetitive regions were excluded. Reference-based SNP calls, including only variant sites where alleles were successfully called in >90% of the isolates, were used to estimate a ML tree of the 44 monophyletic isolates from Banks and Victoria Island muskox carcasses (excluding MX10-3_PSLN, which falls outside this group; Figure 3). This was done in PhyML using the same parameters described above and taking the most closely related isolate (from the Alaska muskox AKM3) as the outgroup for rooting.

### SNP confirmation

To validate SNPs detected among isolates from within a single host based on Illumina sequencing and our bioinformatics pipeline, all unique SNPs (*n* = 5) in the three isolates from muskox “Banks172” were verified using Sanger sequencing. Primers targeting the regions flanking each SNP site were designed using the primer design function in Geneious version 7.1.8 (Kearse et al., 2012; Supplementary Table 2). Each of these five regions was amplified and sequenced in all three isolates. Additionally, to test whether the entire genotyping process (from DNA extraction and sequencing through to SNP calling) is reproducible, as well as to confirm cases wherein highly divergent isolates were obtained from a single animal, technical replicates were performed for four isolates where they were re-cultured from frozen glycerol stock, re-extracted, and re-sequenced during a later run (Table 2).

## Results

### Culture and qPCR

We isolated and sequenced *E. rhusiopathiae* from 39 of 89 carcasses tested from the different populations: 13 of 19 muskoxen sampled on Banks and Victoria Islands, 6 of 23 muskoxen sampled in Alaska, 7 of 16 caribou sampled in BC, 6 of 9 caribou sampled in Alberta, and 7 of 22 moose sampled in BC (Table 1). We sequenced multiple isolates from 18 of these carcasses (Table 2), as well as from the tonsil from a hunter-killed muskox from Victoria Island; this was the only isolate obtained from the 261 hunter-killed animals tested. The proportion of samples that tested positive for *E. rhusiopathiae* using qPCR varied across our sample groups (Table 1). More than 85% of the samples tested from carcasses on Banks and Victoria Islands were qPCR positive, 46% of Alaska muskox samples were qPCR positive (more than were culture positive), and 11% of the caribou and moose samples were qPCR positive (compared to 28% that were culture positive). We did not detect *E. rhusiopathiae* by either qPCR or culture in any of the non-ungulate samples tested (Supplementary Table 1).

### Sequencing

**Figure 4:**
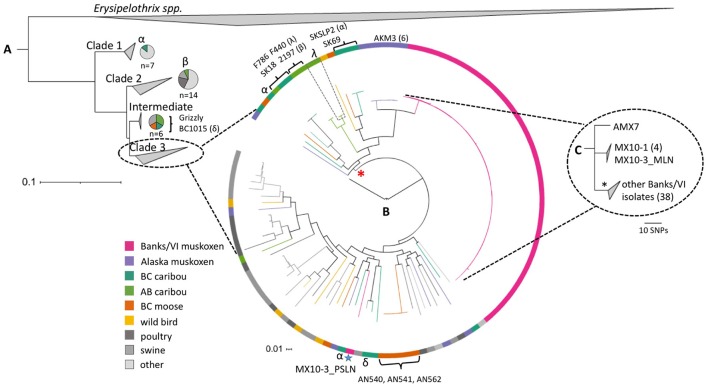
**The diversity of *Erysipelothrix rhusiopathiae* in North American wild ungulates, shown within the broader global population structure. (A)** The phylogenetic tree of the three major clades (collapsed) generated in PhyloPhlAn using >400 conserved bacterial protein sequences, and rooted to other *Erysipelothrix* spp. (*E. tonsillarum* and *E.* sp. strain 2). It comprises one hundred-fifty *E. rhusiopathiae* isolates, including sequences available on GenBank and isolates previously described (Forde et al., 2016). Specific isolates named in this figure are described in Table 2. The species breakdown within Clades 1, 2 and intermediate is shown using pie-charts. **(B)** The relationship among isolates within the dominant Clade 3, shown with a circularized maximum likelihood (ML) tree rooted to the Fujisawa reference genome (intermediate clade). This tree is based on the curated set of core single nucleotide polymorphisms (SNPs) inferred to be outside of recombinant segments by the program Gubbins (Croucher et al., 2015). Colored branches and blocks represent the host species of origin for the various isolates. The blue star indicates the only isolate of the 45 tested from Banks and Victoria Islands that falls outside of the clonal lineage (from the pre-scapular lymph node of MX10-3). Brackets indicate cases of a clone shared between animals. Greek letters (α, β, δ, λ) represent cases of polyclonal infection within individual caribou, with isolates from a single individual shown by the same symbol. The red asterisk indicates the lineage within Clade 3 that contains only isolates from northern wildlife. **(C)** This ML tree generated using reference-based SNP calls shows the relationship among the 44 isolates within the clonal lineage from Banks and Victoria Island muskoxen (shown in greater detail in Figure 3). The asterisk denotes the dominant branch within this group of isolates. VI, Victoria Island; BC, British Columbia; AB, Alberta.

In this study, we found representation across all three phylogenetic clades of *E. rhusiopathiae* (Forde et al., 2016), although with a preponderance of Clade 3 isolates (81/87, Figure 4), which we previously found to be the dominant clade. Figure 4 shows the phylogenetic relationship of ungulate isolates within the broader global collection of isolates previously sequenced from various host species. *Erysipelothrix rhusiopathiae* isolates from all 13 culture-positive muskox carcasses on Banks and Victoria Islands (Table 2, Sample Group 1) belonged to the same monophyletic lineage within Clade 3, with the exception of a single isolate (Figures 4B,C). The two isolates sequenced from the tonsil of an apparently healthy muskox slaughtered during the 2010 community harvest on Victoria Island (VI10-23) also belonged to this same lineage. Within the monophyletic lineage, a maximum of 26 SNP differences separated any pair of isolates from one-another. Three branches stemmed from the inferred common ancestor of this group of isolates (Figure 4C). The isolate from Aulavik National Park on northern Banks Island, AMX7 (Figures 2A, 4C), had nine unique SNPs not found among the other isolates in this lineage. A second branch grouped the four isolates from MX10-1, along with the isolate from the mesenteric lymph node (MLN) of MX10-3; this cluster of isolates all shared eight SNPs that distinguished them from the other two branches within this lineage. Finally, the other 38 isolates from 12 muskoxen on both islands (including two isolates from the lungs of MX10-3 and the isolates from the hunter-killed muskox) clustered within the third branch (Figure 4C, black asterisk). Isolates within this clonal lineage differed among themselves by a maximum of 14 SNPs. One isolate of the 45 isolates sequenced from Banks and Victoria Island muskoxen, cultured from the pre-scapular lymph node (PSLN) of MX10-3 (Figure 2B; blue star in Figure 4), differed from the other isolates by more than 2700 SNPs. Technical replicates performed on both the MLN and PSLN isolates from this animal yielded identical SNP calls as the original samples.

We sequenced 11 isolates from six culture-positive Alaska muskoxen (Sample Group 2), all of which belonged to Clade 3. Within our collection of isolates, the isolates from Alaska muskox AKM3 were the most closely related to the clonal lineage from Banks and Victoria Islands, although they differed from this group of isolates by more than 600 SNPs. Among the six Alaska muskoxen, isolates differed by roughly 1800–2300 SNPs (Figure 4B). Of 13 isolates sequenced from seven caribou sampled in BC (Sample Group 3-i), one isolate belonged to Clade 1 (Figure 4A), one belonged to the intermediate group, while the other 11 fell into Clade 3. We sequenced nine isolates from six caribou from Alberta (Sample Group 3-ii). One isolate belonged to Clade 2, two isolates belonged to the intermediate group, and the remaining six were part of Clade 3. Of the nine isolates that we sequenced from seven moose from BC (Sample Group 3-iii), one belonged to the intermediate group of isolates, while the other eight belonged to Clade 3.

#### Within-host variability

In muskoxen from Banks and Victoria Islands from which multiple isolates were sequenced (*n* = 11), isolates from the same animal differed by a maximum of three SNPs (Figure 3), with the exception of the polyclonal infection in MX10-3 described above. Sanger sequencing confirmed the five SNP differences among the three Banks172 isolates. We sequenced six isolates from one Alaskan muskox, AKM3, among which we found only two variant sites: one SNP was shared by three isolates, while the other was unique to one isolate.

Among caribou from BC, two of three animals from which multiple isolates were sequenced demonstrated polyclonal infection. One case was SKSLP2, a caribou that was sampled the day after it was observed moribund (designated by “α” in Figure 4). One isolate from the lungs of this caribou belonged to Clade 1, with all SNPs confirmed by a technical replicate; two of the four other isolates from SKSLP2 (a second one from the lungs, and one from skeletal muscle) were in phylogenetically distinct parts of Clade 3, while isolates from its bone marrow and liver differed by only one SNP. For caribou 1015, the two bone marrow isolates belonged to Clade 3 and the intermediate clade, respectively, differing by more than 4100 SNP differences (designated by “δ” in Figure 4). For caribou SK18, both bone marrow isolates were identical.

We also observed polyclonal diversity in both Alberta caribou from which multiple isolates were sequenced. The two isolates sequenced from the bone marrow sample of caribou 2197 fell into Clades 2 and 3 (designated by “β” in Figure 4). Three isolates were sequenced from two different bone marrow samples from caribou F440; two isolates were identical (one from each of the samples), while the third isolate differed from the others by 416 SNPs, including the other isolate from the same bone marrow sample (designated by “λ” in Figure 4). Finally, in the cases where two isolates were sequenced from the bone marrow of individual moose, both isolates from AN562 were identical, while there was only one SNP difference between the two isolates from AN541.

#### Strain sharing

In addition to the finding of a clonal lineage of *E. rhusiopathiae* among muskoxen from across Banks and Victoria Islands wherein a limited number of SNPs were detected, we observed five additional cases of *E. rhusiopathiae* clones being shared among animals (Figure 4, shown by brackets). One case involved two isolates in the intermediate clade that differed by a single SNP; these were from a boreal caribou from BC and a central mountain caribou from Alberta (Figure 4A), representing different Designatable Units. The four additional cases of shared clones were among Clade 3 isolates (Figure 4B). First, two isolates from BC caribou SKSLP2 (marrow and liver) clustered with the isolate from caribou SK69; SKSLP2_li was one SNP different from the other two. These two carcasses were found approximately 30 km apart within a month of one-another. Secondly, one of the isolates from F440 was identical to one from 2197; both these caribou come from the Redrock Prairie Creek herd in Alberta. The third case again involved caribou of different DUs: both SK18 boreal caribou isolates were identical to the isolate from the central mountain caribou F786. Finally, five isolates from three different moose (AN540, AN541 and AN562) were identical, with the exception of a single SNP difference in one of the two AN541 isolates. Two of these moose were found dead within the same week at approximately the same location, while the third moose was found 2 months later about 100 km from the first two cases. No strains were shared among the different ungulate species.

## Discussion

Prior to the muskox die-offs on Banks and Victoria Islands (Kutz et al., 2015), *E. rhusiopathiae* had not been implicated in large-scale wildlife mortality events, although individual cases or small clusters of deaths associated with this bacterium had been reported in wild ungulates (Bruner et al., 1984; Campbell et al., 1994). In this study, we isolated *E. rhusiopathiae* from tissues collected from muskoxen, caribou and moose across a broad geographic range where unusual acute mortalities and/or population declines occurred over a common timeframe. This led us to hypothesize that the emergence of a single strain might be responsible for the mortalities. Results from whole genome sequencing of isolates from these different populations, however, refuted this hypothesis, indicating that the geographically disparate ungulate mortalities were not caused by a shared strain of *E. rhusiopathiae*.

We observed three distinct levels of genomic heterogeneity of *E. rhusiopathiae* among the populations in this study. (1) There was minimal heterogeneity among and within most individual muskoxen sampled on Banks and Victoria Islands in the Canadian Arctic Archipelago (Sample Group 1), with only one of 45 isolates differing by more than 26 SNPs, representing a case of polyclonal diversity within an individual. (2) Among Alaska muskoxen (Sample Group 2) there was moderate heterogeneity, with isolates from different individuals all falling within Clade 3 but differing by several hundred SNPs. (3) Caribou and moose in Alberta and BC (Sample Group 3) harbored high diversity, including isolates from multiple clades. This diversity is on a similar level to that seen throughout the entire global collection of *E. rhusiopathiae* isolates from multiple hosts and continents (Figure 4). In this latter group, polyclonal diversity was present within several individual caribou, and we detected some cases wherein a single clone was shared among animals. The varying levels of *E. rhusiopathiae* heterogeneity among the sample groups might reflect differences in (i) the amount of time since the bacterium was introduced into the population, (ii) the underlying transmission dynamics of *E. rhusiopathiae* in these populations, or (iii) the level of existing diversity of this bacterium among the geographically disparate areas, with these explanations not being mutually exclusive.

At least two potential, non-exclusive explanations for the limited diversity observed on Banks and Victoria Islands can be initially considered: exposure to a common and possibly widespread source, or between-animal transmission (Robinson et al., 2013). The use of whole-genome sequencing eliminates poor discriminatory power as a possible explanation for the finding of a common strain. Considering the high degree of mortality and the predominance of a single clonal lineage of *E. rhusiopathiae* across the vast area encompassed by these islands, it is possible that these patterns are associated with a recent introduction of the bacterium into a previously naïve population. We did not find any evidence that this clone was linked to livestock sources or strains circulating at more southern latitudes (Figure 4). Previous research failed to identify a clock-like signal in *E. rhusiopathiae* isolates collected over a timeframe of several decades, so a global substitution rate has not been established for this bacterium (Forde et al., 2016). Therefore, it was not possible to estimate the date of the most recent common ancestor of the isolates from Banks and Victoria Islands using phylogenetic inference. Ongoing testing of archived muskox serum samples may help elucidate historic temporal patterns of exposure to this bacterium on these islands.

The presence of a clonal lineage across both Banks and Victoria Islands suggests that muskoxen on these islands are somehow epidemiologically connected. However, since muskoxen are non-migratory and their movement between these islands and to and from the mainland is uncommon (van Coeverden de Groot, 2001), the spread of *E. rhusiopathiae* across these islands is unlikely to have occurred exclusively through direct transmission. In contrast to swine, where carrier status is believed to be common (20–40%) (Stephenson and Berman, 1978), we found limited evidence that muskoxen act as carriers of the bacterium: of 261 hunter-killed muskoxen sampled, only four were qPCR positive and one subsequently culture positive for *E. rhusiopathiae*. While it is possible that this animal could have subsequently developed disease, this adult male was in good body condition with no gross abnormalities noted during government slaughter inspection associated with the commercial harvest. Environmental survival of *E. rhusiopathiae* is possible for more than 6 months, but its amplification outside of a host is unlikely; early studies monitoring the viability of *E. rhusiopathiae* under different environmental conditions failed to show any evidence of population growth or maintenance (Wood, 1973; Chandler and Craven, 1980). Additionally, the *E. rhusiopathiae* genome lacks several genes for the biosynthesis of essential nutrients for growth (e.g., many amino acids, cofactors, and vitamins) (Ogawa et al., 2011). Given the broad host range of *E. rhusiopathiae*, the maintenance and spread of this bacterium in the Arctic may involve multiple species (Kutz et al., 2015). Species such as polar bears, arctic fox, fish, and migratory birds all range very broadly and could serve as reservoirs and transport hosts, linking the islands and facilitating spread. Similarly, lemmings are cyclically abundant and sympatric with muskoxen (Larter, 1998), and may play a role in maintaining and amplifying the bacteria (Kutz et al., 2015). Although we failed to detect *E. rhusiopathiae* in opportunistic samples tested from non-ungulate species on Banks and Victoria Islands (Supplementary Table 1), the sample size was limited and with the exception of wolf samples, relatively geographically restricted.

The finding of a clonal lineage of *E. rhusiopathiae* within muskoxen on Banks and Victoria islands (Sample Group 1) is in stark contrast with the extensive diversity seen in the other ungulate populations sampled. The high degree of *E. rhusiopathiae* strain diversity found within the smaller geographic areas occupied by Sample Groups 2 and 3 indicates heterogeneous sources, and suggests that *E. rhusiopathiae* may be endemic in these populations. Hence, it is unlikely that ungulate-to-ungulate transmission or a single point source introduction of *E. rhusiopathiae* were major drivers in these mortalities. However, a few nearly identical isolates (differing by a single SNP) occurred among caribou or moose, so exposure to a common source or sporadic cases of between-animal transmission cannot be excluded.

The detection of polyclonal infections has important implications for molecular epidemiological studies. Closely related strains are commonly taken as evidence for potential transmission links (Wylie et al., 2005); however, this study confirmed that such connections may be overlooked if only a single isolate is selected per individual (Döpfer et al., 2008; Ågren et al., 2014). The challenge of detecting mixed infections may soon be partially overcome through the use of novel bioinformatics approaches that do not require multiple isolates to be sequenced (Eyre et al., 2013).

While the mortalities among the different populations, and most mortalities within Sample Groups 2 and 3, do not appear to be epidemiologically linked based on shared *E. rhusiopathiae* strains, they may instead be connected by common changes in exposure or susceptibility to this bacterium. If considered within the framework of the “epidemiological triangle” of agent, host and environment (Wobeser, 2006), all three factors may contribute to the disease outcome. Cumulative stressors, both biotic and abiotic, could be sufficient for tipping the balance in the interaction between host and pathogen from a bacterium that is otherwise tolerated, to one capable of contributing to negative health outcomes (Beldomenico and Begon, 2010; Verbrugghe et al., 2012). Because *E. rhusiopathiae* is a generalist and opportunist bacterium (Brooke and Riley, 1999), common stressors among or within these different populations could allow unrelated strains to opportunistically cause disease and facilitate transmission. If this is true, differences in bacterial heterogeneity observed among the study populations, rather than being associated with different transmission patterns or being the result of newly introduced vs. endemic strains, could instead reflect pre-existing bacterial diversity in these disparate environments, thus representing a third plausible explanation for the limited diversity seen on Banks and Victoria Islands. These islands were glaciated during past glacial maxima and currently harbor substantially lower biodiversity than western Alaska, which was part of the species-rich Beringia during the last ice age (Hoberg et al., 2003), and Alberta and BC, which have comparatively higher species diversity (Kays and Wilson, 2009). It follows that a generalist bacterial species would have considerably more diversity in an environment where a greater diversity and abundance of potential host species exists, compared to an area with a relatively recent history of colonization by a lower diversity and abundance of vertebrate hosts. Thus, although whole genome sequencing enabled us to determine the diversity of *E. rhusiopathiae* in the different ungulate populations, as a stand-alone method it did not allow us to distinguish among the multiple possible explanations for the limited diversity found on Banks and Victoria Islands, nor did it allow us to differentiate between a recently introduced vs. a previously existing pathogen.

It is worth noting that within the global collection of isolates, one branch of Clade 3 which comprises about half of the isolates from caribou, moose and muskoxen, including the predominant Banks and Victoria islands clonal lineage (Figure 4, shown by red asterisk), has not been detected in domestic animals, nor at lower latitudes, possibly suggesting a high latitude or Beringean lineage. Although challenging to predict, both changes in climate and in human and animal contact patterns could result in an expansion of the range of this particular lineage. Meanwhile, the fact that several isolates from northern ungulates, including the single divergent isolate from Victoria Island, belong to lineages of *E. rhusiopathiae* that contain a mix of isolates from wildlife and livestock, suggests that there might be the potential for long-range introductions from distant sources.

The possible role of infectious diseases in widespread wildlife population declines, such as those in barren ground caribou (*Rangifer tarandus groenlandicus*), often remains enigmatic (Vors and Boyce, 2009). There is strong evidence to suggest that septicemia with *E. rhusiopathiae* was the proximate cause of death among the muskoxen on Banks and Victoria Islands (Kutz et al., 2015), and acute death due to septicemia, with few premonitory signs, is among the common disease presentations in swine and other species (Leighton, 2008; Opriessnig and Wood, 2012). It is, therefore, reasonable to hypothesize that the isolation of *E. rhusiopathiae* from the other study populations is associated with the death of the animals, particularly those where there was evidence of acute mortality such as in some of the BC caribou. There is evidence of regular ante-mortem exposure to *E. rhusiopathiae* in muskoxen and caribou based on seroconversion (Kutz, unpublished data). Moreover, of 59 genes we examined that are associated with *E. rhusiopathiae* pathogenicity, 51 were determined to be core genes, supporting the likelihood that the strains isolated from wild ungulates are indeed capable of causing disease (Supplementary Table 3). However, we cannot rule out the possibility that the detection of *E. rhusiopathiae* in tissues sampled from older carcasses could be the result of post-mortem bacterial transmigration, wherein bacteria enter tissues after death (Morris et al., 2006), or contamination from the external environment. Anecdotal reports in some of the older literature suggest that *E. rhusiopathiae* is commonly found in dead and decomposing matter (Klauder, 1938; Shuman, 1970). Longitudinal epidemiological studies that involve targeted surveillance and timely sample collection and testing will be necessary to better understand the role of *E. rhusiopathiae* in wild ungulate mortalities and investigate what other factors may be contributing, including viral infections, poisoning, and/or environmental stressors. A further limitation of this study, although a reality for many wildlife studies, was the fact that samples were collected opportunistically through different health surveillance initiatives, resulting in inconsistencies in the type of samples collected and in storage conditions. We found the agreement between culture and qPCR to be inconsistent for samples tested using both methods (Table 1). Factors such as long-term sample storage (Eriksson et al., 2014), suboptimal transport conditions, bacterial concentration and viability, or the presence of inhibitory substances for qPCR could all differentially influence the sensitivity of culture and qPCR. Finally, the sensitivity of qPCR and culture for detecting asymptomatic carriers is unknown. Where possible, we based the choice of tissues for sampling on literature pertaining to asymptomatic carriage/infection of *E. rhusiopathiae* in livestock; however, these may not be the most relevant samples for other potential carriers, including different wildlife species.

In conclusion, the use of bacterial genomics allowed us to determine that geographically widespread mortalities associated with *E. rhusiopathiae* in northern wild ungulates were not linked by a common bacterial strain. Instead, the underlying epidemiology was found to be complex, with three distinct patterns of bacterial genetic heterogeneity, which may reflect either different underlying transmission dynamics, the amount of time the bacterium has been present in these environments, or geographic differences in the diversity of this bacterium. While the limited diversity of *E. rhusiopathiae* on Banks and Victoria Islands initially suggested the introduction of a new bacterium with subsequent host-to-host transmission, the availability of genomic data from other ungulate populations provided a valuable point of comparison, leading us to consider alternative explanations. The detection of various strains of *E. rhusiopathiae* in similar northern wild ungulate mortalities over a common timeframe may point to a broader issue of reduced population resilience or environmental changes favoring bacterial amplification. This finding warrants a return to the classical epidemiological triangle to examine underlying stressors, including climate change, that could make hosts more permissive to opportunistic pathogens (Kutz et al., 2015). Understanding pathogen emergence and disease dynamics in boreal and arctic environments will require investigations that look beyond single factors and simple answers.

## Data accessibility

Raw sequence reads are available on GenBank as part of BioProject PRJNA288715.

## Author contributions

Conceived and designed the study: TF, SK, KO, RZ, RB, JD, BM, and FV. Coordinated the study: SK, KO, and TF. Performed sample collections and provided associated metadata: LA, SC, TD, MD, BE, LF, BM, CN, AN, SS, HS and SK. Performed laboratory work and data analysis: TF. Drafted the paper: TF. Edited and contributed to the manuscript: SK, KO, RZ, RB, JD, FV, LA, SC, MD, LF, CN, and HS. All authors read and approved the final manuscript.

## Funding

Funding for this research was provided by the Natural Sciences and Engineering Research Council of Canada Discovery Grant and Northern Supplement and Canada Graduate Scholarship, University of Calgary Eyes High, Izaak Walton Killam Pre-Doctoral Scholarship, Canada North Outfitters, Nunavut General Monitoring Program, Nunavut Harvesters Association, Inuvialuit Implementation, BC Oil and Gas Research and Innovation Society (OGRIS), Weyerhaeuser Co. Ltd, Alberta Environment and Parks Governments of Northwest Territories and Nunavut, and fRIResearch. Any opinions, findings and conclusions expressed herein are those of the authors and do not necessarily reflect the views of funding entities. Funding entities had no involvement in the design of the study or in the interpretation of results.

### Conflict of interest statement

The authors declare that the research was conducted in the absence of any commercial or financial relationships that could be construed as a potential conflict of interest. Any use of trade, firm, or product names is for descriptive purposes only and does not imply endorsement by any Canadian institution or the U.S. Government.

## Supplementary material

Supplementary File 1 **Methods: samples tested for *Erysipelothrix rhusiopathiae* from non-ungulate species**.

Supplementary File 2 **Detection of *Erysipelothrix tonsillarum* in ungulate samples**.

Supplementary Table 1 **Results of samples from non-ungulate species tested for *Erysipelothrix rhusiopathiae***.

Supplementary Table 2 **Primers used for Sanger sequencing confirmation of unique single nucleotide polymorphisms found in different isolates from Banks Island muskox 172**.

Supplementary Table 3 **Presence of genes associated with *Erysipelothrix rhusiopathiae* pathogenicity among the isolates included in this study**.
