# Supplementary material for: Bacterial Genomics Reveal the Complex Epidemiology of an Emerging Pathogen in Arctic and Boreal Ungulates
Source: Front Microbiol. 2016 Nov 7;7:1759. doi: 10.3389/fmicb.2016.01759 (PMC5097903; doi:10.3389/fmicb.2016.01759)
Supplement: Supplementary File 1 — Methods: samples tested for Erysipelothrix rhusiopathiae from non-ungulate species. [file DataSheet1.DOCX]

**Supplementary File 1. Methods: samples tested for *E. rhusiopathiae* from non-ungulate species**

As a preliminary investigation into the host range of *E. rhusiopathiae* on Banks and Victoria Islands, samples were opportunistically collected from species sympatric to muskoxen on these islands and on the nearby mainland. Swabs were taken from the cloaca of hunted snow geese (*Chen caerulescens*; *n* = 88) collected on western Banks Island in June 2014. Swabs were placed on Amies gel transport medium (VWR International, Edmonton, Alberta, Canada) and maintained at ambient temperature on Banks Island for up to two weeks until they could be frozen, shipped and stored at -20°C for three months prior to testing. Swabs of the mucous exterior of arctic char (*Salvelinus alpinus*, *n* = 73) were collected from four different rivers on Victoria Island between July and September, 2013, as well as from one arctic char and one trout in Aulavik National Park on Banks Island in July, 2013. These were placed on transport medium and stored refrigerated or frozen until being shipped and tested within four months of collection. Tonsils from 115 wolves (*Canis lupus*) collected by local trappers between 2010 and 2013 (50 from Banks Island, 35 from Victoria Island, and 30 from the Sahtu region of mainland Northwest Territories) were tested. The intact wolf heads were stored frozen until the time the tonsils were removed and shipped for testing. Finally, the entire digestive system from two North American brown lemmings (*Lemmus trimucronatus*) trapped near Sachs Harbour on Banks Island were also tested. qPCR was performed on DNA extracted directly from wolf tonsils (Table S1). Lemming samples were tested using both direct PCR and culture. Swabs from geese and fish were cultured in a similar manner to tissue samples: the tip of the swab was placed in 3 ml of BHI selective broth with 5% serum and incubated for 48 hours at 37°C with 5% CO_2_. The swab was then used to streak a CA plate.
