# Supplementary material for: Bacterial Genomics Reveal the Complex Epidemiology of an Emerging Pathogen in Arctic and Boreal Ungulates
Source: Front Microbiol. 2016 Nov 7;7:1759. doi: 10.3389/fmicb.2016.01759 (PMC5097903; doi:10.3389/fmicb.2016.01759)
Supplement: Supplementary File 2 — Detection of Erysipelothrix tonsillarum in ungulate samples. [file DataSheet2.DOCX]

**Supplementary File 2. Detection of *Erysipelothrix tonsillarum* in ungulate samples**

In the interest of identifying isolates that tested qPCR negative for *E. rhusiopathiae* despite having the correct colony morphology, these isolates were subsequently tested using an *E. tonsillarum*-specific probe. Two caribou samples that were PCR negative for *E. rhusiopathiae* had colony morphology consistent with *Erysipelothrix* spp., which subsequently tested positive for *E. tonsillarum* using the species-specific PCR. Three samples (two from Alaska muskoxen and the sample from MX10-4 on Victoria Island) were PCR positive for *E. rhusiopathiae* and had colony morphology consistent with *Erysipelothrix* spp., but the individual isolates tested negative for *E. rhusiopathiae* on qPCR; these were subsequently identified as *E. tonsillarum*. Finally, both *E. rhusiopathiae* and *E. tonsillarum* were identified by species-specific qPCR in some cases when multiple colonies were selected from the original blood agar plate for isolation (AKM3, SK133, 2197, Grizzly)*.*

It is impossible to conjecture whether the culture of *E. tonsillarum* from samples from the various wild ungulate populations, including in co-infection with *E. rhusiopathiae*, is significant. The vast majority of *E. tonsillarum* strains are believed to be non-pathogenic for most species (Harada et al., 2011; Takahashi et al., 1994, 1996, 2008). However, *E. tonsillarum* has been shown to be pathogenic in dogs (Takahashi et al., 1993), and was occasionally isolated from chronic lesions in swine at slaughter where *E. rhusiopathiae* was not isolated (Bender et al., 2011). Its pathogenic potential in wild ungulates may require further consideration.

**References**

Bender, J. S., Irwin, C. K., Shen, H.-G., Schwartz, K. J., and Opriessnig, T. (2011). *Erysipelothrix* spp. genotypes, serotypes, and surface protective antigen types associated with abattoir condemnations. *J. Vet. Diagn. Investig.* 23, 139–142.

Harada, K., Muramatsu, M., Suzuki, S., Tamura, Y., Sawada, T., and Takahashi, T. (2011). Evaluation on the pathogenicity of *Erysipelothrix tonsillarum* for pigs by immunosuppression with cyclophosphamide or dexamethasone. *Res. Vet. Sci.* 90, 20–22. doi:10.1016/j.rvsc.2010.05.009

Takahashi, T., Fujisawa, T., Umeno, A., Kozasa, T., Yamamoto, K., and Sawada, T. (2008). A taxonomic study on *Erysipelothrix* by DNA-DNA hybridization experiments with numerous strains isolated from extensive origins. *Microbiol. Immunol.* 52, 469–478. doi:10.1111/j.1348-0421.2008.00061.x

Takahashi, T., Nagamine, N., Kijima, M., Suzuki, S., Takagi, M., Tamura, Y., et al. (1996). Serovars of *Erysipelothrix* strains isolated from pigs affected with erysipelas in Japan. *J. Vet. Med. Sci.* 58, 587–589.

Takahashi, T., Takagi, M., Yamaoka, R., Ohishi, K., Norimatsu, M., Tamura, Y., et al. (1994). Comparison of the pathogenicity for chickens of *Erysipelothrix rhusiopathiae* and *Erysipelothrix tonsillarum*. *Avian Pathol.* 23, 237–245. doi:10.1080/03079459408418992

Takahashi, T., Tamura, Y., Yoshimura, H., Nagamine, N., Kijima, M., Nakamura, M., et al. (1993). *Erysipelothrix tonsillarum* isolated from dogs with endocarditis in Belgium. *Res. Vet. Sci.* 54, 264–265.
