# Supplementary material for: Bacterial Genomics Reveal the Complex Epidemiology of an Emerging Pathogen in Arctic and Boreal Ungulates
Source: Front Microbiol. 2016 Nov 7;7:1759. doi: 10.3389/fmicb.2016.01759 (PMC5097903; doi:10.3389/fmicb.2016.01759)
Supplement: Supplementary Table 1 — Results of samples from non-ungulate species tested for Erysipelothrix rhusiopathiae. [file Table1.DOCX]

**Supplementary Table 1. Results of samples from non-ungulate species tested for *Erysipelothrix rhusiopathiae.***

| **Species** | **Location** | **Sample type** | **Collection date** | **# Samples tested** | **# direct PCR positive** | **# Culture positive** |
| --- | --- | --- | --- | --- | --- | --- |
| geese | Banks Island | cloacal swabs; hunted birds | June 2014 | 88 | - | 0/88 |
| fish | Victoria Island  Banks Island | swabs of mucosal surface; fresh-caught fish | Jul-Sep 2013  Jul 2013 | 73  2 | - | 0/73  0/2 |
| wolves | Banks Island  Victoria Island  Sahtu | tonsils;  trapped animals | 2011-2013  2010-2012  2011-2013 | 50  35  30 | 0/115 | - |
| lemmings | Banks Island | digestive tract; trapped animals | 2014 | 2 | 0/2 | 0/2 |
