# Supplementary material for: Bacterial Genomics Reveal the Complex Epidemiology of an Emerging Pathogen in Arctic and Boreal Ungulates
Source: Front Microbiol. 2016 Nov 7;7:1759. doi: 10.3389/fmicb.2016.01759 (PMC5097903; doi:10.3389/fmicb.2016.01759)
Supplement: Supplementary Table 2 — Primers used for Sanger sequencing confirmation of unique single nucleotide polymorphisms found in different isolates from Banks Island muskox 172. [file Table2.DOCX]

**Supplementary Table 2. Primers used for Sanger sequencing confirmation of unique single nucleotide polymorphisms (SNPs) found in different isolates from Banks Island muskox 172.** Positions are with respect to the *Erysipelothrix rhusiopathiae* Fujisawa reference genome [GenBank: NC_015601].

| **SNP site** | **Forward Primer** | **Reverse primer** | **Region Amplified** |
| --- | --- | --- | --- |
| Banks172ma_249413 | TGGTGCAGGATGAGGATTACC | CCAAGTCGCTCTAACGAAGC | 249317-249458 |
| Banks172ma_717828 | AGCGGCTACGATTCACTCTG | ATCACCCACAAACAGCAGTT | 717704-717887 |
| Banks172ma_1495440 | AAGAAGTCCCAGAAGTGCCG | CCACGCACGGTTGATGTTTT | 1495362-1495489 |
| Banks172sp-i_339071 | TTGGGACACGTCTTGCGAAT | ACCACCGATTACCATCACAACA | 338995-339101 |
| Banks172sp-i_1208468 | AGAAGCGTTCGGTCATGTGT | ACACCAATGCATCTGTTGTCTG | 1208297-1208490 |
